# The cerebellum in migraine: a narrative review of anatomical, imaging, and clinical evidence

**DOI:** 10.3389/fneur.2026.1877070

**Published:** 2026-10-05

**Authors:** Ruonan Zhang, Jiali Huang, Xiaoping Fan, Wanqun Hu, Liang Dong, Jiying Zhou

**Affiliations:** 1 Department of Neurology, The First Affiliated Hospital of Chongqing Medical University, Chongqing, China; 2 School of Nursing, Chongqing Medical University, Chongqing, China; 3 Phase I Clinical Research Center, The First Affiliated Hospital of Chongqing Medical University, Chongqing, China; 4 Department of Neurology, Chongqing Jiulongpo District Science City People's Hospital, Chongqing, China

**Keywords:** cerebellar imaging, cerebellum, CGRP, medication-overuse headache, migraine, nociception, pain modulation, vestibular migraine

## Abstract

Migraine is usually framed through trigeminovascular pathways and interactions among the brainstem, hypothalamus, and cortex. The cerebellum has received less attention, although human studies place it within circuits that process trigeminal nociception and vestibular signals. This narrative review examines anatomical and experimental work together with imaging, genetic, and clinical studies, giving greatest weight to direct human migraine evidence. Repeated-measures imaging suggests that cerebellar connectivity changes across the migraine cycle, but the cohorts are small. Structural findings vary with analytic method, and population based testing does not show a general cerebellar deficit in common migraine. Evidence is more coherent in defined contexts: trigeminal stimulation recruits lobules VI/VIIIa and Crus I, vestibular migraine involves posterior cerebellar and wider vestibular-sensory networks, and treatment imaging shows cerebellar changes without localizing the site of CGRP-directed action. Findings in medication-overuse headache and familial hemiplegic migraine provide additional state-related and mechanistic clues. Taken together, the cerebellum is best viewed as a modulatory component of distributed migraine networks, with possible roles in sensory gain and vestibular integration. It is neither a demonstrated attack generator nor a validated treatment target. The next step is to test whether within-person cerebellar measures reproduce across attacks and whether intervention changes a prespecified circuit-level endpoint.

## 1 Introduction

Migraine is a common primary headache disorder characterized by recurrent moderate-to-severe headache with photophobia, phonophobia, nausea, or vomiting (1). It remains a leading cause of neurological disability (2). Its clinical course spans prodrome, aura, headache, and postdrome, with symptom profiles and neural systems varying across stages (3).

Conceptual models of migraine pathophysiology have moved from vascular explanations toward broader network-based understanding. Wolff-era vascular theory emphasized intracranial and extracranial vascular changes as the dominant mechanism of attacks, but subsequent imaging and experimental work showed that vascular alterations alone cannot account for the complexity and variability of the migraine phenotype (4). Current models generally regard migraine as a disorder involving dysfunction across multiple interacting brain systems, including cortical hyperexcitability, cortical spreading depolarization, activation of the trigeminovascular system, and central sensitization (5, 6).

Within this broader network framework, the cerebellum has remained peripheral in migraine research. This partly reflects the older view of the cerebellum as a structure devoted mainly to motor coordination (7). In headache research, Vincent and Hadjikhani (8) were among the first to discuss its possible relevance to migraine in a dedicated manner. More broadly, cerebellar pain research has moved from passive co-activation toward active modulation (9). The motor centered view has also narrowed: the cerebellum is now recognized as a contributor to cognition, emotion, sensory integration, prediction, and timing (10, 11).

Non-motor cerebellar neuroscience and recent imaging have renewed interest in this question (12–17). Earlier reviews established the biological case; here, the emphasis is on what individual studies can support about migraine specificity, attack state, and treatment-related change (14, 17).

We ask whether reported cerebellar findings are specific to migraine, whether they vary with attack state or persist over time, and whether they justify therapeutic targeting. Human migraine studies carry the main evidential weight; other sources are used when they clarify a mechanism or define a limit.

## 2 Scope and approach

We conducted a narrative review of studies relevant to cerebellar involvement in migraine. PubMed, Web of Science, Google Scholar, and reference lists were searched from database inception to April 2026, and PubMed was updated through August 26, 2026. Search terms paired migraine with cerebellum or cerebellar imaging and with structural or functional connectivity, nociception, trigeminal pathways, medication overuse, CGRP, familial hemiplegic migraine, vestibular migraine, visual snow, TMS, or tDCS. The PubMed update screened titles and abstracts published from January 2024 onward using migraine with cerebellum or cerebellar; forward and backward citation tracking was conducted from key reviews and foundational studies (8, 9, 14–17). We gave priority to peer-reviewed human studies with cerebellar endpoints, especially longitudinal, randomized, or population-based work, and retained negative or non-convergent findings to reduce selective emphasis. Experimental and adjacent-condition studies were included selectively when they clarified mechanisms or boundaries. Case reports and small series were retained only when they captured ictal physiology or rare mechanisms unavailable in larger studies. Study selection was not performed by duplicate independent reviewers, and no formal risk-of-bias assessment or effect-size pooling was undertaken. Table 1 summarizes each study's relevance to migraine and principal interpretive limitation.

**Table 1:** Selected human studies of cerebellar findings relevant to migraine.

Domain	Study	Sample	Method	Cerebellar or relevant negative finding	Relevance to migraine and main limitation
General nociception	Moulton et al. (9)	56 fMRI/PET studies	ALE meta-analysis	Consistent activation in vermal lobules IV/V, bilateral lobule VI, and bilateral Crus I.	Contextual evidence; not migraine-specific.
Trigeminal nociception	Mehnert et al. (23)	54 participants plus 18 validation participants	Trigeminal chemosensory fMRI	Activity in ipsilateral VI/VIIIa, Crus I, and vermal VIIIa; VIIIa signal correlated with pain ratings.	Contextual human evidence; no migraine cohort.
Trigeminocervical nociception	Basedau et al. (41)	25 exploratory plus 36 independent participants	Trigeminal and greater occipital nerve fMRI	Broad, somatotopically differentiated cerebellar responses.	Contextual human evidence; healthy cohorts.
Migraine-cycle dynamics	Stankewitz et al. (43)	12 patients; 82 sessions	Repeated pCASL and hypothalamic connectivity	Hypothalamic connectivity with cerebellar and other regions was lowest during headache and increased toward the next attack.	Direct repeated-measures evidence; small cohort.
Migraine-cycle dynamics	Esteves et al. (44)	10 patients, four migraine phases; 14 controls, two menstrual phases	Repeated resting-state fMRI	Peri-ictal network changes were widespread, with many altered edges involving the cerebellum.	Direct repeated-measures evidence; small menstrual migraine cohort.
Pain-evoked response	Schwedt et al. (46)	24 episodic migraine; 27 controls	Pain-evoked fMRI	Cerebellar activation occurred in both groups; migraine greater-than-control regions were elsewhere.	Direct migraine comparison; no cerebellar hyperresponsiveness relative to controls.
Resting-state connectivity	Ke et al. (50)	39 episodic migraine without aura; 35 controls	Degree centrality and seed connectivity	Lower left Crus I centrality and weaker Crus I-default-mode connectivity.	Direct cross-sectional evidence; no validated biomarker.
Resting-state connectivity	Chanraud et al. (47)	17 MOH, 18 episodic migraine, 17 controls; smaller FC subsets	VBM and precuneus-seed fMRI	Lower precuneus connectivity with left lobules IV-V in MOH than episodic migraine.	Direct MOH evidence; dependence interpretation correlational.
Phase-group connectivity	Cao et al. (48)	34 interictal, 10 acute-attack, 32 controls	VBM and seed connectivity	Higher left frontal-lobule VI connectivity in the acute-attack group than controls.	Direct phase-group evidence; not within-person change.
Aura-specific connectivity	Gollion et al. (49)	21 migraine with aura, 12 without aura, 18 controls	Insula seed-to-voxel fMRI	Antero-dorsal insula coupling with upper cerebellar clusters overlapping vermis VI in migraine with aura.	Direct subtype evidence; modest sample.
Functional metaanalysis	Chen et al. (51)	Systematic review and ALE meta-analysis	ReHo meta-analysis	Stable convergence was in brainstem and thalamus, not the cerebellum.	Negative meta-analytic finding; study availability and methods remain heterogeneous.
Structural metaanalysis	Jia and Yu (53)	8 structural studies	Coordinate-based meta-analysis	Lower gray matter in the right cerebellar culmen.	Positive meta-analytic signal; small, older evidence base.
Structural metaanalysis	Masson et al. (54)	New 19/19 cohort plus coordinate meta-analysis	VBM and meta-analysis	No consistent gray- or white-matter volume or cortical thickness difference.	Negative meta-analytic finding; method and study availability remain limiting.
Structural metaanalysis	Zhang et al. (55)	40 studies; 1,616 migraine and 1,681 controls	AES-SDM and ALE	Lower bilateral hemispheric lobule IX with AES-SDM; no matching ALE convergence.	Method-dependent meta-analytic evidence.
Structural metaanalysis	Al Qawasmeh et al. (56)	845 migraine; 656 controls	ALE meta-analysis	Lower cerebellar declive gray matter in the migraine-with aura subgroup.	Subgroup-specific evidence.
Population morphometry	Palm-Meinders et al. (59)	84 migraine; 35 controls	Population-based cross-sectional VBM	No clear cerebellum-specific difference.	Population-based negative finding; this VBM analysis was not longitudinal.
Primary morphometry	Zhang et al. (57)	32 migraine without aura; 32 controls	SBM, VBM, and DTI	Higher gray matter in bilateral anterior vermis, lobules I-V.	Direct cross-sectional evidence.
Primary morphometry	Mehnert and May (58)	54 migraine and matched controls	Cerebellum-focused VBM	Higher gray matter in right posterior cerebellar regions.	Direct cross-sectional evidence; direction differs from some meta-analyses.
Pediatric morphometry	Aytaç et al. (60)	74 adolescents; 70 controls	Retrospective volumetry and cortical thickness	Lower bilateral lobule VI volume and thinner right Crus I/II.	Pediatric cross-sectional evidence; adult generalization uncertain.
Longitudinal morphometry	Messina et al. (61)	Baseline 73/46; follow-up 24/25	VBM over approximately 4 years	Lower cerebellar gray matter at baseline; longitudinal changes were mainly cortical.	Longitudinal human evidence; no clear cerebellar progression.
Outcome-associated structure	Liu et al. (62)	56 high-frequency migraine; 37 controls	VBM, covariance, 2-year follow-up	Greater baseline cerebellar volume in the poorer-outcome subgroup; covariance related to later headache frequency.	Longitudinal association; independent prognostic validity remains unknown.
Treatmentassociated structure	Rosell-Mirmi et al. (63)	Baseline 189 migraine and 63 controls; paired MRI in 101 treated patients (43 erenumab, 58 onabotulinumtoxinA)	Prospective 6-month MRI	Different erenumab and onabotulinumtoxinA trajectories included bilateral cerebellar cortical volume differences.	Treatment-associated change; mechanism and site of action unresolved.
Microstructure	Qin et al. (64)	VBM 50/50; DTI 46/46	SUIT-VBM and DTI	Lower FA in vermis VI extending to lobules V/VI; higher diffusivity in right inferior cerebellar peduncle and spinal trigeminal nucleus.	Direct cross-sectional microstructural evidence.
Structural connectome	Matoso et al. (65)	14 migraine without aura; 15 controls, all female	Interictal diffusion and T1 connectomics	Greater connectivity, global efficiency, and posterior node degree.	Direct evidence; small interictal all-female cohort.
Multimodal coupling	Li et al. (66)	36 chronic, 94 episodic, 51 controls	Structural-functional coupling	Differences in cerebellum 3 and vermis 7 in chronic migraine; vermis 7 also differed in episodic migraine.	Direct cross-sectional evidence; clinical correlation not definitive.
Clinical neurophysiology	Brighina et al. (69)	10 migraine with aura; 7 controls	Paired-pulse cerebellar TMS	Reduced cerebellar inhibition of contralateral motor cortex.	Small mechanistic study; not a treatment trial.
Cerebellar learning	Gerwig et al. (70)	32 women with migraine; 32 controls	Conditioned eyeblink acquisition	Lower acquisition; corrected subgroup signal clearest in migraine with aura.	Task-specific behavioral evidence; female cohort.
Broad cerebellar function	Koppen et al. (71)	200 common migraine, 82 controls, 13 FHM1 positive controls	Motor, learning, visuospatial, and balance battery	No overall common-migraine deficit; marked impairment in FHM1.	Population-based negative finding; separates common migraine from FHM1.
Posterior circulation infarct like lesions	Kruit et al. (67)	295 migraine; 140 controls	Population-based MRI	Posterior-circulation infarct-like lesions were more common in migraine (5.4%) than controls (0.7%), with most in cerebellar border-zone regions.	Population-based vascular evidence; subclinical lesions are not a functional syndrome.
Medication overuse headache	Fumal et al. (74)	16 MOH; 68 healthy controls	FDG-PET before and after withdrawal	Vermal hypermetabolism moved toward control levels after withdrawal.	Small longitudinal MOH evidence; no episodic migraine comparator.
Medication overuse headache	Liu et al. (77)	27 MOH; 17 controls	FDG-PET with 1-year outcome	Cerebellar hypermetabolism and covariance were strongest in the poor-outcome subgroup.	Outcome association; small subgroup-defined cohort.
CGRP-treatment imaging	Ziegeler et al. (83)	27 migraine	Trigeminal fMRI before and after erenumab	Lower activation in bilateral cerebellar clusters and other central regions.	Uncontrolled treatment imaging; site of action uncertain.
CGRP-treatment imaging	Filippi et al. (84)	61 randomized; 44 protocol complete	Placebo-controlled crossover resting-state fMRI	Treatment differences involved cerebellar, thalamic, and PAG networks.	Randomized imaging; carry-over required first-period comparison.
CGRP-treatment imaging	Basedau et al. (85)	44 randomized; 40 scanned	Placebo-controlled trigeminal fMRI	Weighted treatment interaction involved thalamus, operculum, and putamen, not the cerebellum.	Randomized imaging; no cerebellar cluster in the weighted treatment interaction.
Vestibular migraine	Shin et al. (90)	2 cases	Ictal/interictal FDG-PET	Bilateral cerebellar hypermetabolism during attacks within a broader network.	Direct ictal evidence; two-case series.
Vestibular migraine	Xiong et al. (91)	57 vestibular migraine; 88 controls	Dynamic ALFF	Lower activity dynamics in the right posterior cerebellum.	Direct cross-sectional evidence; metric-specific.
Vestibular migraine	Chen et al. (92)	55 vestibular migraine; 57 controls	Morphological connectivity	A reduced network component included cerebellar regions.	Direct network evidence; clinical correlations did not survive correction.

AES-SDM, anisotropic effect-size seed-based d mapping; ALE, activation likelihood estimation; ALFF, amplitude of low-frequency fluctuation; DTI, diffusion tensor imaging; FA, fractional anisotropy; FDG, fluorodeoxyglucose; fMRI, functional magnetic resonance imaging; FHM1, familial hemiplegic migraine type 1; MOH, medication-overuse headache; PAG, periaqueductal gray; pCASL, pseudo-continuous arterial spin labeling; ReHo, regional homogeneity; SBM, surface-based morphometry; SUIT, spatially unbiased infratentorial template; VBM, voxel-based morphometry.

## 3 Anatomical and physiological basis

### 3.1 Cerebellar non-motor organization and pain-related topography

The cerebellum lies in the posterior fossa and consists of cerebellar cortex, deep cerebellar nuclei, and three pairs of cerebellar peduncles (7). Its cortical microcircuit is highly regular: mossy fibers and climbing fibers form the two major input systems; granule cells relay mossy fiber input via parallel fibers; and Purkinje cells serve as the sole output neurons of the cerebellar cortex, projecting inhibitory GABAergic signals to the deep nuclei (7). Excitatory outputs from the deep nuclei then reach the thalamus, red nucleus, and multiple brainstem structures via the superior and inferior cerebellar peduncles (18). This uniform microarchitecture has often been taken to imply that the cerebellum performs related computational operations across different functional domains, including motor, cognitive, affective, and sensory processing.

The traditional tripartite scheme divides the cerebellum into vestibulocerebellar, spinocerebellar, and cerebrocerebellar territories, corresponding broadly to balance and eye movements, sensorimotor control, and higher cognitive functions (10). However, this framework is now recognized as incomplete. Using resting-state functional connectivity, Buckner et al. showed that cerebellar organization mirrors large-scale cerebral cortical networks in a far more differentiated manner than older motor-centered models had implied (12). In this scheme, anterior lobe regions and lobule VIII are preferentially linked to sensorimotor networks, whereas Crus I/II and posterior lobule VII show stronger relationships with higher-order frontoparietal and associative systems (12). This network-based view provides anatomical plausibility for cerebellar participation in pain, prediction, attention, salience, and multisensory processing in migraine.

Pain-related topography within the cerebellum adds to this anatomical case. Functional MRI work by Welman et al. (19) showed somatotopic, rather than diffuse, cerebellar responses to nociceptive stimulation: noxious stimulation of the thumb was associated mainly with activity in ipsilateral lobule VI and contralateral Crus II, whereas toe stimulation preferentially engaged bilateral lobules VIII-IX and IV-V. These nociceptive representations partly overlapped with, but were not identical to, those elicited by non-nociceptive somatosensory input (16, 19). This pattern suggests that the cerebellum may process nociceptive and non-nociceptive signals through partially distinct microcircuits.

### 3.2 Nociceptive and trigeminal pathways reaching the cerebellum

At least three major pathways can convey nociceptive information to the cerebellum (9, 16, 20). One is the spino-olivo-cerebellar route, which is closely associated with the climbing fiber system. Signals from C-fiber nociceptors can ascend through postsynaptic dorsal column pathways to the inferior olive and then reach cerebellar cortex via climbing fibers (21). Because climbing fibers generate powerful complex spikes in Purkinje cells and are often viewed as teaching or error-related signals, this pathway has been proposed to transmit temporally precise information relevant to adaptive responses to injury or threat (9, 22). Classic animal work by Ekerot et al. (21, 22) provided direct evidence that C-fiber nociceptive input can activate cerebellar climbing fiber systems in cats.

A second pathway is the spino-reticulo-cerebellar route, which reaches the cerebellum through mossy fiber inputs via reticular and pontine relays (9, 20). Compared with climbing fibers, mossy fibers distribute information more broadly through granule cells and parallel fibers and are thought to encode aspects of context, spatial organization, and signal magnitude. In nociceptive settings, this system may therefore provide a different informational stream from that carried by climbing fibers, potentially combining injury-related input with state-dependent sensorimotor and autonomic signals (9).

For migraine, trigeminal inputs are especially relevant. The spinal trigeminal nucleus receives sensory input from craniofacial structures and the meninges and sends direct and indirect projections toward the cerebellum (6). Mehnert et al. (23) used intranasal ammonia as a trigeminal chemosensory nociceptive stimulus and demonstrated significant activation in ipsilateral lobules VI and VIIIa, Crus I, and vermal lobule VIIIa, together with enhanced functional communication between the cerebellum and brainstem nuclei. These findings show that the human cerebellum participates in trigeminal nociceptive processing; their relevance to migraine remains inferential.

Visceral nociceptive work provides a weaker extension. Saab and Willis showed that noxious colorectal distension modulates posterior-vermis Purkinje cell activity and engages lateral medullary reticular neurons projecting to the vermis (20). These data extend cerebellar nociceptive access beyond somatic inputs, although their migraine relevance is indirect.

### 3.3 Connectivity with pain-modulatory, vestibular, and hypothalamic-autonomic networks

The cerebellum is connected, directly or indirectly, with several core nodes of pain processing and pain modulation (9, 16). One such node is the periaqueductal gray (PAG), a major center in descending pain modulation (24). Animal and human data suggest functional relationships between the cerebellar vermis or fastigial nucleus and the PAG in pain-related contexts (9, 25, 26). In humans, Borsook et al. (25) observed concurrent activation of the vermis and PAG during noxious brushing and heat stimulation, which supports co-engagement but not by itself a directional regulatory pathway. In animals, Cerminara et al. (26) showed that stimulation of the ventrolateral PAG can suppress nociceptive input reaching the cerebellum, suggesting that PAG-cerebellar circuitry may participate in adaptive pain-related behavior. Frontera et al. (27) further demonstrated in mice that excitatory fastigial projections to the ventrolateral PAG can bidirectionally regulate conditioned fear memory, a cross-domain extension suggesting that cerebellar-PAG interactions may also involve affective dimensions of aversive processing.

The thalamus is another important relay. Outputs from deep cerebellar nuclei project to thalamic nuclei, which in turn connect to sensorimotor, prefrontal, insular, and cingulate cortices (18). In experimental work, stimulation of the lateral cerebellar nucleus modulated nociceptive coding in intralaminar thalamic nuclei, particularly the parafascicular complex (28). Because these thalamic nuclei relay information to areas involved in salience, interoception, and affective-motivational pain processing, cerebellar influence at this level could be relevant to the way migraine-related sensory signals are integrated and interpreted (29).

Connections with prefrontal, cingulate, and insular systems provide a plausible route for cerebellar influence on cognitive-affective and salience-related aspects of migraine. Tract tracing studies have shown closed-loop relationships between the cerebellum and dorsolateral prefrontal cortex (30), a region implicated in attention, cognitive evaluation of pain, and placebo-related analgesia (31). Functional relationships with the anterior cingulate cortex have also been reported, consistent with a possible role in pain-related salience and emotional appraisal (32). Posterior cerebellar territories, especially Crus I/II and lobule VII, are therefore better viewed as part of distributed control and associative networks than as purely vestibular or motor regions.

Other subcortical relays remain more speculative in migraine. For example, the zona incerta can modulate precerebellar structures and has been implicated in central pain, but direct migraine evidence is currently limited (33, 34).

The cerebellum also sits at the intersection of vestibular, autonomic, and hypothalamic systems. This anatomical position is relevant to dizziness, disequilibrium, nausea, yawning, attentional fluctuation, and affective discomfort, all of which may accompany migraine. The PAG and related brainstem systems coordinate emotional-motor and autonomic responses, providing another route through which cerebellar-brainstem interactions may influence non-headache symptom expression (35). These connections support a network-level role, but they do not make any one symptom cerebellum-specific.

### 3.4 Experimental evidence that cerebellar stimulation can modulate nociception

A further line of evidence comes from animal stimulation studies, which provide causal rather than purely correlational support for cerebellar nociceptive modulation (9). In squirrel monkeys, Siegel and Wepsic (36) found that electrical stimulation of the intermediate part of the anterior lobe increased nociceptive thresholds to tail shock. In rats, Saab and Willis (37) observed that chemical stimulation of cerebellar cortex enhanced visceral nociceptive reflexes, whereas stimulation of the fastigial nucleus reduced them. These opposing effects have been interpreted within a model in which cerebellar cortex and deep nuclei exert functionally distinct influences: increased cortical inhibitory output may suppress deep nuclear activity and thereby reduce descending antinociceptive effects, whereas reduced cortical inhibition may disinhibit deep nuclei and strengthen endogenous pain suppression (9).

Derived from non-migraine experiments, this model provides a conceptual account rather than direct migraine evidence. It suggests that cerebellar involvement in pain extends beyond passive co-activation and that different subregions may contribute in directionally different ways. In migraine, a context-dependent modulatory role is therefore more plausible than a unitary attack generator.

These anatomical and experimental observations make cerebellar participation in migraine related processes biologically plausible. The unresolved questions concern the disease states, patient subsets, and pathways through which that participation becomes clinically meaningful.

### 3.5 Human evidence in migraine

#### 3.5.1 Cerebellar activation during acute nociceptive stimulation

Healthy-volunteer nociceptive studies characterize the general cerebellar pain substrate; migraine-specific inference requires patient cohorts, trigeminal stimulation, or migraine-like attacks. Casey et al. (38) reported increased blood flow in the cerebellar vermis during repeated noxious thermal stimulation in a PET study published in 1994, representing one of the first direct imaging observations to associate the cerebellum with pain processing. Subsequent PET and functional MRI studies confirmed cerebellar recruitment during acute nociceptive stimulation (9, 16).

Meta-analytic evidence supports the reproducibility of these findings. In a 2010 meta-analysis of 56 fMRI and PET studies, Moulton et al. identified consistent activation in vermal lobules IV/V, bilateral lobule VI, and bilateral Crus I during acute noxious stimulation (9). Additional work by Helmchen et al. (39, 40) suggested that cerebellar responses are not identical for innocuous and noxious thermal stimuli. In their studies, nociceptive-specific activation was most prominent in the deep cerebellar nuclei, the anterior vermis, and bilateral lobule VI (39, 40). They also observed stronger activation in hemispheric lobules III-VI in individuals reporting greater pain intensity, providing one of the earliest links between cerebellar nociceptive activity and subjective pain perception (40).

Cerebellar activity during pain also extends beyond the sensory consequences of stimulation. As discussed above, Welman et al. (19) showed that the cerebellar representation of pain expectation only partly overlaps with that of actual nociceptive input. Noxious thumb stimulation preferentially engaged ipsilateral lobule VI and contralateral Crus II, whereas expectation related overlap was more prominent in bilateral Crus I and lobule VI (19). This distinction is relevant to migraine because anticipation, uncertainty, and sensory prediction are clinically salient features of the disorder and could involve cerebellar computations beyond sensory mapping alone.

The trigeminal system is particularly important in this context. Mehnert et al. (23) found nociception-related activity in ipsilateral lobules VI and VIIIa, Crus I, and vermal VIIIa; signal strength in the prominent VIIIa response correlated with pain ratings. The study also showed stronger communication within the cerebellum and between cerebellar and brainstem regions (23). Basedau et al. (41) subsequently mapped nociceptive input from trigeminal branches and the greater occipital nerve in an exploratory cohort of 25 healthy volunteers and an independent cohort of 36, with broad but somatotopically differentiated cerebellar responses. Together, these studies establish a human trigemino-cerebellar substrate, although neither enrolled patients with migraine.

#### 3.5.2 Functional dynamics across the migraine cycle

Migraine is a cyclic disorder, and its temporal structure matters when interpreting cerebellar findings. Whether cerebellar involvement occurs primarily before attacks, during the headache phase, or during recovery remains uncertain, but recent longitudinal imaging has made the question more tractable.

Dense-sampling studies first established that hypothalamic-brainstem coupling changes over the migraine cycle. In one patient scanned daily for 30 days and three spontaneous attacks, Schulte and May (42) reported phase-related hypothalamic coupling with the spinal trigeminal nucleus and dorsal rostral pons, but not a cerebellar result. Direct cerebellar evidence came from Stankewitz et al. (43), who studied 12 patients across 82 sessions. Hypothalamic connectivity with sensory and frontal cortices, basal ganglia, and cerebellar regions was lowest during headache and increased over the pain-free interval toward the next attack. Esteves et al. (44) later scanned 10 patients with menstrual or menstrual-related migraine in four migraine phases and 14 controls in two menstrual phases. Connectivity became less individually stable before attacks, and ictal and postictal increases involved several networks, with many altered edges including the cerebellum. These cohorts support cycle-related cerebellar network dynamics, but their size and restricted phenotypes preclude a triggering or causal interpretation.

Other imaging studies of spontaneous or provoked attacks have mainly emphasized the brainstem and hypothalamus. Afridi et al. (45), for example, used glyceryl trinitrate to provoke migraine-like headache and highlighted lateralized brainstem activation rather than a discrete cerebellar signal. The available studies are compatible with dynamic cerebellar participation, but do not yet define its timing or mechanistic role.

#### 3.5.3 Interictal resting-state abnormalities

Resting-state fMRI studies suggest that cerebellar function may differ even outside overt headache attacks, but the literature is heterogeneous. Broadly, the findings fall into two groups: altered connectivity between the cerebellum and cortical or subcortical regions, and altered local spontaneous activity measures within the cerebellum itself.

Connectivity studies have used several designs. In Schwedt et al. (46), painful stimulation activated the cerebellum in both episodic migraine and control groups; the regions with significantly greater activation in migraine lay elsewhere in the pain network. This study therefore shows shared cerebellar nociceptive engagement, not cerebellar hyperresponsiveness. Chanraud et al. (47) found lower connectivity between a left-precuneus seed and left cerebellar lobules IV-V in medication-overuse headache than in episodic migraine, alongside broader default-mode and memory-network changes; the dependence-related interpretation was correlational. Cao et al. (48) used an unpaired cross-sectional comparison of interictal, acute attack, and control groups. The acute-attack group showed greater connectivity between the left lower middle frontal cortex and left cerebellar lobule VI than controls, but the design did not establish within-person state change (48). For migraine with aura, Gollion et al. (49) found interictal coupling between bilateral antero-dorsal insula seeds and upper cerebellar clusters overlapping vermis VI; this pattern was not reported in migraine without aura in the same study. Ke et al. (50) examined 39 patients with episodic migraine without aura and 35 controls at rest, finding lower degree centrality in left Crus I and weaker connectivity between Crus I and default-mode regions. The direction and specificity of these findings vary with paradigm, phase group, aura status, and clinical context.

Local activity measures are even less consistent. Current meta-analytic evidence does not support a stable or reproducible decrease in cerebellar regional homogeneity. In an ALE meta-analysis, Chen et al. (51) mainly identified increased regional homogeneity in the brainstem and left thalamus; cerebellar changes were not among the most stable replicated findings. Studies using ALFF and fALFF have reported abnormal spontaneous activity in multiple cerebellar regions (52), but both increases and decreases have been described. Differences in migraine subtype, attack frequency, scanner timing, preprocessing, and analytical choices likely contribute to this variability.

#### 3.5.4 Structural findings: gray matter, white matter, and infratentorial observations

Structural MRI provides a mixed picture, and the disagreement is visible even between meta-analyses. Jia and Yu (53) pooled eight structural studies and reported lower gray matter in the right cerebellar culmen. A later coordinate-based meta-analysis found no consistent gray- or white-matter volume or cortical-thickness difference (54). In a 40-study analysis, Zhang et al. (55) found lower bilateral hemispheric lobule IX with AES-SDM, whereas ALE identified no corresponding cerebellar cluster. An earlier ALE study reported lower declive gray matter only in the migraine-with-aura subgroup (56). The analyses used different datasets and algorithms, often with overlapping primary studies, so their divergent results do not represent independent replications.

Primary studies are similarly variable. Increased volume has been reported in the anterior vermis and in posterior regions including lobule VI, VIIIa, and Crus I/II (57, 58), whereas Palm-Meinders et al. (59) found no clear cerebellum-specific difference in a population-based cross-sectional VBM analysis. The CAMERA-2 cohort was longitudinal at the epidemiological level, but this VBM analysis was cross-sectional because comparable baseline T1 imaging was unavailable. In adolescents, a 2026 retrospective study reported lower bilateral lobule VI volume and thinner right Crus I/II in 74 patients with migraine without aura relative to 70 controls (60). That finding broadens the age range of reported abnormalities; whether the same structural pattern occurs in adults is unknown.

Longitudinal studies do not resolve the direction. Messina et al. (61) observed lower cerebellar gray matter at baseline in 73 patients, but the changes detected in the 24 patients rescanned after approximately 4 years were mainly cortical. In high-frequency migraine, Liu et al. (62) found greater baseline bilateral cerebellar volume in patients with a poorer 2-year outcome and an association between right VIIIa-cuneus structural covariance and later headache frequency. Whether these baseline measures predict outcome in an independent cohort is unknown. Rosell-Mirmi et al. (63) enrolled 189 people with migraine and 63 controls at baseline; 101 treated patients completed paired MRI at 6 months, including 43 who received erenumab and 58 who received onabotulinumtoxinA. The treatment groups had different structural trajectories, including lower bilateral cerebellar cortical volume at follow-up in the erenumab group. The comparison does not establish whether this change was a direct drug effect, a consequence of clinical improvement, or another form of plasticity.

Microstructural and connectome studies add further variation. Qin et al. (64) reported lower fractional anisotropy in vermis VI extending into bilateral lobules V/VI, together with higher axial, mean, and radial diffusivity in the right inferior cerebellar peduncle and higher diffusivity in the spinal trigeminal nucleus. Matoso et al. (65) examined 14 women with interictal episodic migraine without aura and 15 controls and found greater structural connectivity, global efficiency, and posterior cerebellar node degree in the migraine group. In a 2026 multimodal study of 36 chronic, 94 episodic, and 51 control participants, structural-functional coupling differed in cerebellum 3 and vermis 7 in chronic migraine; vermis 7 also differed in episodic migraine (66). These cross-sectional findings support network involvement but do not identify a single direction of cerebellar pathology.

Posterior circulation and infratentorial lesions form a separate evidence stream. In the population-based CAMERA studies, migraine was associated with more posterior circulation infarct-like lesions and infratentorial hyperintensities (67, 68). Such lesions indicate vascular or infratentorial vulnerability, not a uniform functional cerebellar syndrome.

#### 3.5.5 Clinical neurophysiology and cerebellar function

A paired-pulse TMS study found reduced cerebellar inhibition of contralateral motor cortex in 10 patients with migraine with aura compared with seven controls (69). Gerwig et al. (70) found reduced acquisition of conditioned eyeblink responses in 32 women with migraine relative to 32 controls; the corrected subgroup signal was clearest in migraine with aura. A broader population sample did not show a generalized deficit. Koppen et al. (71) tested 200 people with common migraine and 82 controls across motor, learning, visuospatial, and balance tasks and found no overall group difference, despite a higher prevalence of posterior cerebellar ischemic lesions in migraine. The same battery did identify marked impairment in 13 participants with CACNA1A-related familial hemiplegic migraine. Cerebellar differences in common migraine may therefore be task- or phenotype-specific.

#### 3.5.6 Methodological heterogeneity and state-trait interpretation

Some heterogeneity is biological: migraine subtype, attack phase, disease duration, attack frequency, medication use, and psychiatric comorbidity alter the sampled state. Study size and analytic choices introduce separate variation, particularly preprocessing, cerebellar normalization and segmentation, and metric selection. In resting-state and morphometric work, whole-brain rather than cerebellum-specific normalization may determine whether a small cluster is recovered. Seed choice and the use of local activity, VBM, SUIT-VBM, DTI, or graph metrics further limit direct comparison.

The state-trait distinction helps organize this variability. Repeated-measures and phase-group studies address attack-related dynamics (43, 44, 48), whereas structural and microstructural studies are better suited to questions of stable vulnerability or accumulated burden (53, 56–59, 61, 62, 64, 65, 72, 73). Longitudinal outcome associations have not yet established prognostic validity (62, 63). Reversible vermal metabolism after medication withdrawal may reflect drug exposure, chronic pain adaptation, or partial recovery (74). Any proposed biomarker should therefore specify whether it is expected to vary across the migraine cycle, remain stable within individuals, or predict a prespecified clinical outcome.

### 3.6 Special mechanistic and clinical contexts

#### 3.6.1 Medication-overuse headache

Medication-overuse headache (MOH) provides one of the more clinically informative contexts in which cerebellar abnormalities have been described (75). Fumal et al. (74) used 18F-FDG PET in 16 patients with chronic migraine and analgesic overuse and 68 healthy controls. Before withdrawal, patients showed vermal hypermetabolism together with lower metabolism in orbitofrontal, thalamic, and anterior cingulate regions (74). 3 weeks after withdrawal, vermal metabolism moved toward control levels, whereas orbitofrontal changes persisted (74). There was no episodic-migraine comparator. The reversible cerebellar signal may therefore reflect medication exposure, chronic pain adaptation, or compensation rather than a feature specific to MOH.

Texture analysis has also identified vermal abnormalities in MOH (76), although their cellular basis and direction of effect cannot be inferred from MRI texture. In a recent PET follow-up study of 27 patients and 17 controls, cerebellar hypermetabolism and stronger cerebellar metabolic covariance were most evident in the poor-outcome subgroup at one year (77). The sample was small and subgroup-defined; prognostic use would require prospective validation in an independent cohort. Chronic pain-related plasticity provides another non-specific explanation (78). These abnormalities involve more than the cerebellum. Orbitofrontal and anterior cingulate changes implicate valuation and control, while Chanraud et al. (47) reported connectivity compatible with dependence-related processes (74). Cerebellar change should therefore be tested against medication exposure, headache burden, affective symptoms, and recovery within the same patients rather than assigned to pathology or compensation from cross-sectional data.

#### 3.6.2 CGRP-related observations

CGRP biology has transformed migraine therapeutics (13, 79). Preclinical anatomical studies identify CGRP and CGRP-receptor components in cerebellar circuits, particularly Purkinje cells, CGRP-positive afferent fibers, and the medial cerebellar nucleus; the distribution varies by component and species (15, 79). Expression establishes anatomical plausibility but does not show that cerebellar signaling mediates migraine or treatment response.

Pharmacological efficacy does not identify its anatomical site of action. In rats, peripherally administered galcanezumab reached the cerebellum at concentrations far below those in plasma (80), consistent with the limited blood-brain barrier penetration of monoclonal antibodies (81). Low cerebellar exposure neither excludes downstream cerebellar effects nor supports a cerebellar explanation for non-response. The same caution applies in the opposite direction: central imaging changes after treatment do not demonstrate direct receptor engagement in the region where they are observed.

Small-molecule CGRP receptor antagonists may enter the central nervous system more readily, but this does not localize their clinical effect to the cerebellum (82). In an uncontrolled before and-after fMRI study, erenumab treatment was followed by reduced trigeminal-stimulation responses in the thalamus, operculum, and bilateral cerebellar clusters; the changes were interpreted as central consequences that could arise downstream of peripheral modulation (83). Randomized imaging studies provide a stricter test. In RESET BRAIN, erenumab and placebo differed in resting-state networks involving the cerebellum, thalamus, and periaqueductal gray, although carry-over required the main comparison to be restricted to the first treatment period (84). By contrast, a placebo-controlled trigeminal fMRI study found its weighted treatment interaction in the thalamus, operculum, and putamen rather than the cerebellum (85). A prospective cohort also found different cerebellar structural trajectories after erenumab and onabotulinumtoxinA (63). These observations show that cerebellar measures can change during treatment; none identifies the cerebellum as the site of therapeutic action.

#### 3.6.3 Familial hemiplegic migraine as a genetic clue

Familial hemiplegic migraine (FHM) offers some of the strongest genetic clues linking migraine biology to the cerebellum (8). FHM1 is caused by CACNA1A mutations affecting the alpha1A subunit of the P/Q-type calcium channel, which is highly expressed in Purkinje cells (86). Approximately 40%−50% of families with CACNA1A-FHM have cerebellar signs ranging from nystagmus to usually mild, late-onset progressive ataxia (87). CACNA1A mutations also underlie episodic ataxia type 2 and spinocerebellar ataxia type 6, creating clinical overlap between migraine and cerebellar channelopathies (86, 88). FHM2 and FHM3 involve ATP1A2 and SCN1A, respectively. Both genes are expressed in the cerebellum, but the clinical association with cerebellar dysfunction is clearest for CACNA1A (8). A concise genephenotype summary is provided in Table 2.

**Table 2:** Familial hemiplegic migraine subtypes and cerebellar-relevant genetic or phenotypic features.

Subtype	Causative gene/ locus	Encoded protein/function	Cerebellar-relevant expression	Clinical phenotype and disease spectrum
FHM1	CACNA1A (19p13)	Cav2.1 P/Q-type voltage-gated calcium channel alpha1A subunit; regulates synaptic vesicle release	Highly expressed in Purkinje cells; involved in climbing fiber-Purkinje cell synaptic transmission	Migraine with hemiplegic aura; approximately 40%-50% of CACNA1A-FHM families have cerebellar signs ranging from nystagmus to usually mild, late-onset progressive ataxia (87). CACNA1A variants also cause episodic ataxia type 2 and spinocerebellar ataxia type 6 (86, 88).
FHM2	ATP1A2 (1q23)	Na+/K+-ATPase alpha2 subunit; maintains neuronal-glial ionic gradients and regulates glutamate clearance	Astrocyte-predominant expression in the adult brain; no established cerebellar enrichment	Migraine with hemiplegic aura; impaired consciousness, seizures, and fever associated severe attacks have been reported, whereas persistent cerebellar signs are not a defining feature (8, 87).
FHM3	SCN1A (2q24)	Nav1.1 voltage-gated sodium channel alpha subunit; regulates neuronal excitability	Expressed in the cerebellum; cell-type relevance to FHM3 remains uncertain	Migraine with hemiplegic aura; SCN1A variants are also associated with epilepsy syndromes, including Dravet syndrome, whereas cerebellar involvement is not a consistent defining phenotype (8, 87).
Shared mechanism	Channelopathy	Ion channel or transporter dysfunction causing excitatory-inhibitory imbalance	Cerebellar cortex and deep cerebellar nuclei	The three FHM subtypes indicate that ion channel dysfunction can appear together with migraine phenotypes and, particularly in CACNA1A-FHM, cerebellar signs. Experimental FHM models support cortical hyperexcitability; direct cerebellar cortical spreading depolarization in human migraine remains unproven (8, 88).

CSD, cortical spreading depolarization; EA2, episodic ataxia type 2; FHM, familial hemiplegic migraine; SCA6, spinocerebellar ataxia type 6.

Perturbations of ion channels or transporters can produce both migraine phenotypes and cerebellar signs, consistent with a shared excitatory-inhibitory imbalance (8, 88). Experimental FHM1 models support increased susceptibility to cortical spreading depolarization (88), but cerebellar cortical spreading depolarization has not been demonstrated in human migraine. FHM is therefore a mechanistic analogy for common migraine, not direct evidence that the same process occurs in both.

#### 3.6.4 Vestibular migraine and non-headache symptom domains

Vestibular migraine is one of the most clinically intuitive settings in which cerebellar relevance is often discussed (8, 89). Patients experience episodic or chronic vestibular symptoms such as spontaneous vertigo, positional vertigo, visually induced dizziness, and postural unsteadiness (89). Because the cerebellum, especially vestibulocerebellar and posterior vermal regions, is central to vestibular integration, vestibular migraine has often been viewed as a natural clinical window into cerebellar mechanisms. Current evidence, however, does not justify treating vestibular migraine as a simple “cerebellar subtype” of migraine.

Structural and functional imaging in vestibular migraine supports a distributed vestibularsensory network. In a small ictal/interictal FDG-PET case series (*n* = 2), Shin et al. (90) observed increased bilateral cerebellar metabolism during attacks, together with changes in frontal, temporal, insular, thalamic, and occipital regions. Xiong et al. (91) found lower dynamics of spontaneous activity in the right posterior cerebellum in 57 patients relative to 88 controls. In a separate sample of 55 patients and 57 controls, Chen et al. (92) identified a reduced morphological-connectivity component that included cerebellar regions; the reported clinical correlations did not survive correction for multiple testing. MRI findings outside the cerebellum, including parieto-insular vestibular cortex and somatosensory connectivity changes (93), further argue against treating vestibular migraine as an isolated cerebellar disorder.

#### 3.6.5 Cognitive-affective, autonomic, and visual-network boundary conditions

Migraine commonly involves more than head pain. Cognitive complaints, attentional fluctuation, affective discomfort, sensory aversion, nausea, yawning, and autonomic changes all point to systems that integrate interoceptive state, salience, prediction, and behavioral control. Cerebellar involvement in these domains is plausible because posterior cerebellar regions interact with prefrontal and associative networks, while the vermis and fastigial circuitry interact more closely with brainstem-autonomic and affective-motor systems (10–12, 27, 30, 35).

The insula-cerebellum coupling reported in migraine with aura provides one human example linking upper cerebellum and interoceptive-salience circuitry (49). Repeated-measures studies suggest that cerebellar network participation can vary with the migraine cycle (43, 44), while MOH studies place cerebellar signals alongside frontal control and dependence-related networks (47, 74). Direct evidence linking these network patterns to cognitive, affective, or autonomic symptoms is still absent.

Visual snow syndrome provides a useful boundary condition. Voxel-based morphometry has shown increased gray matter in visual cortices, V5, and the left cerebellar Crus I/lobule VI region (94). A separate study found altered connectivity from cerebellar seeds in 24 patients and 24 controls, but the cerebellar-seed clusters did not remain significant after adjustment for migraine (95). Together, these findings do not establish a migraine-specific cerebellar abnormality (96).

### 3.7 Working model and translational implications

#### 3.7.1 A network model of cerebellar involvement in migraine

The available evidence places the cerebellum within migraine networks but not at their center. Its contribution appears to vary with disease phase, symptom domain, and phenotype, which is more consistent with a modulatory node than a unitary attack generator. Figure 1 summarizes this working model.

**Figure 1:**
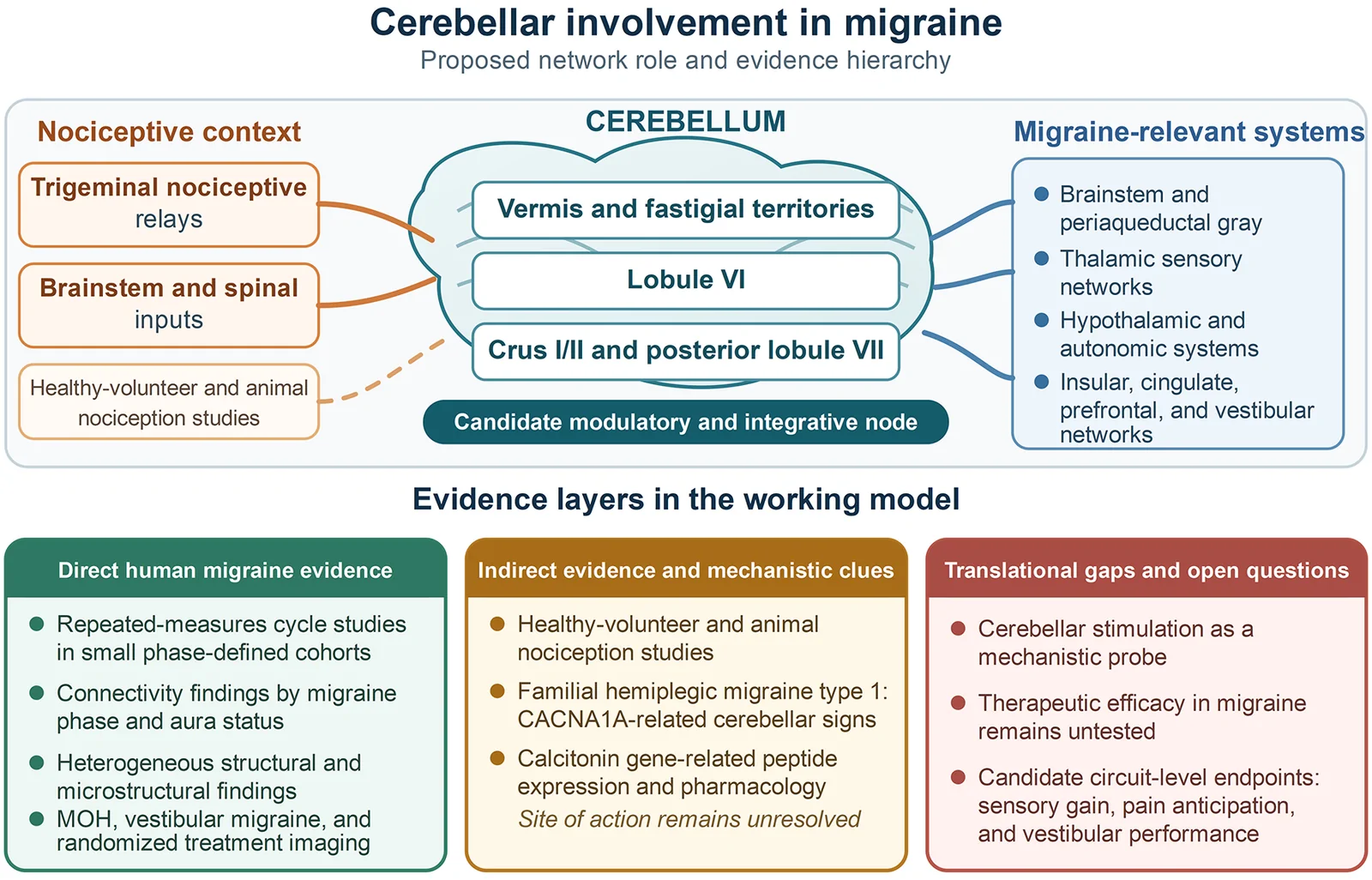
Working model of cerebellar involvement in migraine. The upper panel situates the cerebellum among nociceptive relays and migraine-relevant brainstem, thalamic, hypothalamic, vestibular, and cortical systems. Connector lines indicate anatomical or functional relationships considered in the model; they do not encode direction or causality. The lower panel separates direct human migraine observations from indirect mechanistic evidence and unresolved translational questions. Direct human migraine observations include small repeated-measures studies, connectivity findings by migraine phase or aura status, heterogeneous structural and microstructural results, randomized treatment imaging, and limited studies of medication overuse headache or vestibular migraine. Indirect evidence comes from healthy-volunteer and animal nociception studies, familial hemiplegic migraine type 1 channelopathy, and cerebellar calcitonin gene-related peptide expression. Cerebellar stimulation is currently a mechanistic probe; its therapeutic efficacy in migraine has not been tested. The cerebellum is therefore presented as a candidate modulatory and integrative node, not as a proven migraine-specific generator. MOH, medication-overuse headache.

Within this model, nociceptive input reaches cerebellar circuits through trigeminal, spinal, and brainstem pathways, while cerebellar output is embedded in PAG, thalamic, cortical, vestibular, and hypothalamic systems. A regional pattern is beginning to emerge. Vermal and fastigial territories are most relevant to nociceptive integration, autonomic regulation, and affective motor responses. Lobule VI appears repeatedly in trigeminal, vestibular, and insular connectivity observations. Crus I/II and posterior lobule VII fit better with prediction, attention, and higher-order multisensory control. Whether these regional roles hold within migraine specific circuits remains uncertain.

#### 3.7.2 Neuromodulation and current translational limits

Interest in cerebellar neuromodulation has grown because the cerebellum is accessible to noninvasive stimulation and connected to several migraine-relevant networks (17). In healthy volunteers, cerebellar theta-burst stimulation altered motor-cortical excitability (97). A small paired-pulse TMS study in migraine with aura also found reduced cerebellar inhibition of contralateral motor cortex (69), but this measured circuit physiology rather than treatment efficacy. Non-invasive stimulation can therefore probe cerebellar-cortical physiology, while its analgesic and therapeutic effects in migraine remain untested.

Migraine tDCS trials have mainly targeted visual or primary motor cortex rather than the cerebellum (98–100). Cerebellar stimulation remains an experimental probe, with no established target, dose, or clinical indication in migraine.

## 4 Future directions

Repeated-measures imaging should establish whether cerebellar signals recur within the same person across complete migraine cycles. In MOH, withdrawal studies can separate changes that follow medication exposure from those that track headache burden or clinical recovery. Placebo-controlled treatment imaging should test whether cerebellar change mediates response or simply accompanies reduced peripheral input.

Multimodal phenotyping is also needed. Imaging should be paired with prespecified neuropsychological tasks and measures of affective symptoms. Sensory hypersensitivity, vestibular performance, medication use, and headache burden should be collected in the same participants to determine whether a cerebellar signal follows headache burden or a broader migraine phenotype.

Cerebellar stimulation studies need a prespecified circuit-level endpoint before headache outcomes are interpreted. Sensory gain, pain anticipation, vestibular performance, and cerebellar-brain inhibition are plausible candidates. Across these designs, attack phase and medication exposure should be documented, cerebellum-specific normalization should be used, and the principal result should be replicated in an independent cohort.

## 5 Conclusion

Current evidence places the cerebellum within migraine-related nociceptive and vestibular networks, but not as a demonstrated attack generator. The strongest human evidence concerns trigeminal engagement, phase-dependent connectivity in small repeated-measures cohorts, and selected vestibular findings. Treatment studies show cerebellar imaging change without locating the therapeutic site of action. Structural results remain method-dependent, and population-based functional testing argues against a generalized cerebellar deficit in common migraine.

The model yields two different predictions. Observational studies should find within-person cerebellar measures that covary with prespecified sensory or vestibular states and replicate across attacks. Intervention studies should show that changing a cerebellar circuit alters the corresponding physiological endpoint before a claim is made about headache prevention. Neither prediction has yet been met well enough for a clinical biomarker or therapeutic recommendation.

## Data Availability

The original contributions presented in the study are included in the article/supplementary material, further inquiries can be directed to the corresponding authors.

## References

[1] Headache Headache Classification Committee of the International Headache Society (IHS) The International Classification of Headache Disorders 3rd edition. Cephalalgia. (2018) 38:1–211. doi: 10.1177/0333102417738202 29368949

[2] GBD 2016 Headache Collaborators. Global, regional, and national burden of migraine and tension-type headache, 1990-2016: a systematic analysis for the Global Burden of Disease Study 2016. Lancet Neurol. (2018) 17:954–76. doi: 10.1016/S1474-4422(18)30322-3 30353868 PMC6191530

[3] Goadsby PJ Holland PR Martins-Oliveira M Hoffmann J Schankin C Akerman S. Pathophysiology of migraine: a disorder of sensory processing. Physiol Rev. (2017) 97:553–622. doi: 10.1152/physrev.00034.2015 28179394 PMC5539409

[4] Olesen J Burstein R Ashina M. Origin of pain in migraine: evidence for peripheral sensitisation. Lancet Neurol. (2009) 8:679–90. doi: 10.1016/S1474-4422(09)70090-0 19539239

[5] Brennan KC Pietrobon D. A systems neuroscience approach to migraine. Neuron. (2018) 97:1004–21. doi: 10.1016/j.neuron.2018.01.029 29518355 PMC6402597

[6] Burstein R Noseda R Borsook D. Migraine: multiple processes, complex pathophysiology. J Neurosci. (2015) 35:6619–29. doi: 10.1523/JNEUROSCI.0373-15.2015 25926442 PMC4412887

[7] Purves D Augustine GJ Fitzpatrick D Hall WC LaMantia AS Mooney RD . Neuroscience. 6th ed. Sunderland, MA: Sinauer Associates (2018). ISBN: 9781605353807.

[8] Vincent M Hadjikhani N. The cerebellum and migraine. Headache. (2007) 47:820–33. doi: 10.1111/j.1526-4610.2006.00715.x 17578530 PMC3761082

[9] Moulton EA Schmahmann JD Becerra L Borsook D. The cerebellum and pain: passive integrator or active participator? Brain Res Rev. (2010) 65:14–27. doi: 10.1016/j.brainresrev.2010.05.005 20553761 PMC2943015

[10] Manto M Bower JM Conforto AB Delgado-García JM da Guarda SN Gerwig M . Consensus paper: roles of the cerebellum in motor control-the diversity of ideas on cerebellar involvement in movement. Cerebellum. (2012) 11:457–87. doi: 10.1007/s12311-011-0331-9 22161499 PMC4347949

[11] Schmahmann JD. The cerebellum and cognition. Neurosci Lett. (2019) 688:62–75. doi: 10.1016/j.neulet.2018.07.005 29997061

[12] Buckner RL Krienen FM Castellanos A Diaz JC Yeo BT. The organization of the human cerebellum estimated by intrinsic functional connectivity. J Neurophysiol. (2011) 106:2322–45. doi: 10.1152/jn.00339.2011 21795627 PMC3214121

[13] Edvinsson L Haanes KA Warfvinge K Krause DN. CGRP as the target of new migraine therapies - successful translation from bench to clinic. Nat Rev Neurol. (2018) 14:338–50. doi: 10.1038/s41582-018-0003-1 29691490

[14] Noseda R. Cerebro-cerebellar networks in migraine symptoms and headache. Front Pain Res. (2022) 3:940923. doi: 10.3389/fpain.2022.940923 35910262 PMC9326053

[15] Wang M Tutt JO Dorricott NO Parker KL Russo AF Sowers LP. Involvement of the cerebellum in migraine. Front Syst Neurosci. (2022) 16:984406. doi: 10.3389/fnsys.2022.984406 36313527 PMC9608746

[16] Li CN Keay KA Henderson LA Mychasiuk R. Re-examining the mysterious role of the cerebellum in pain. J Neurosci. (2024) 44:e1538232024. doi: 10.1523/JNEUROSCI.1538-23.2024 38658164 PMC11044115

[17] Prati JM Gianlorenco AC. A new vision of the role of the cerebellum in pain processing. J Neural Transm. (2025) 132:537–46. doi: 10.1007/s00702-024-02872-1 39798004

[18] Schmahmann JD. From movement to thought: anatomic substrates of the cerebellar contribution to cognitive processing. Hum Brain Mapp. (1996) 4:174–98. doi: 10.1002/(SICI)1097-0193(1996)4:3<174::AID-HBM3>3.0.CO;2-0 20408197

[19] Welman FHSM Smit AE Jongen JLM Tibboel D van der Geest JN Holstege JC. Pain experience is somatotopically organized and overlaps with pain anticipation in the human cerebellum. Cerebellum. (2018) 17:447–60. doi: 10.1007/s12311-018-0930-9 29480507 PMC6028829

[20] Saab CY Willis WD. The cerebellum: organization, functions and its role in nociception. Brain Res Brain Res Rev. (2003) 42:85–95. doi: 10.1016/S0165-0173(03)00151-6 12668291

[21] Ekerot CF Garwicz M Schouenborg J. Topography and nociceptive receptive fields of climbing fibres projecting to the cerebellar anterior lobe in the cat. J Physiol. (1991) 441:257–74. doi: 10.1113/jphysiol.1991.sp018750 1816375 PMC1180197

[22] Ekerot CF Oscarsson O Schouenborg J. Stimulation of cat cutaneous nociceptive C fibres causing tonic and synchronous activity in climbing fibres. J Physiol. (1987) 386:539–46. doi: 10.1113/jphysiol.1987.sp016550 3681719 PMC1192478

[23] Mehnert J Schulte L Timmann D May A. Activity and connectivity of the cerebellum in trigeminal nociception. Neuroimage. (2017) 150:112–8. doi: 10.1016/j.neuroimage.2017.02.023 28192274

[24] Ossipov MH Dussor GO Porreca F. Central modulation of pain. J Clin Invest. (2010) 120:3779–87. doi: 10.1172/JCI43766 21041960 PMC2964993

[25] Borsook D Moulton EA Tully S Schmahmann JD Becerra L. Human cerebellar responses to brush and heat stimuli in healthy and neuropathic pain subjects. Cerebellum. (2008) 7:252–72. doi: 10.1007/s12311-008-0011-6 18418691

[26] Cerminara NL Koutsikou S Lumb BM Apps R. The periaqueductal grey modulates sensory input to the cerebellum: a role in coping behaviour? Eur J Neurosci. (2009) 29:2197–206. doi: 10.1111/j.1460-9568.2009.06760.x 19453624

[27] Frontera JL Baba Aissa H Sala RW Mailhes-Hamon C Georgescu IA Léna C . Bidirectional control of fear memories by cerebellar neurons projecting to the ventrolateral periaqueductal grey. Nat Commun. (2020) 11:5207. doi: 10.1038/s41467-020-18953-0 33060630 PMC7566591

[28] Liu FY Qiao JT Dafny N. Cerebellar stimulation modulates thalamic noxious-evoked responses. Brain Res Bull. (1993) 30:529–34. doi: 10.1016/0361-9230(93)90079-Q 8457903

[29] Craig AD. How do you feel? Interoception: the sense of the physiological condition of the body. Nat Rev Neurosci. (2002) 3:655–66. doi: 10.1038/nrn894 12154366

[30] Kelly RM Strick PL. Cerebellar loops with motor cortex and prefrontal cortex of a nonhuman primate. J Neurosci. (2003) 23:8432–44. doi: 10.1523/JNEUROSCI.23-23-08432.2003 12968006 PMC6740694

[31] Tracey I Mantyh PW. The cerebral signature for pain perception and its modulation. Neuron. (2007) 55:377–91. doi: 10.1016/j.neuron.2007.07.012 17678852

[32] Vogt BA. Pain and emotion interactions in subregions of the cingulate gyrus. Nat Rev Neurosci. (2005) 6:533–44. doi: 10.1038/nrn1704 15995724 PMC2659949

[33] Bhuvanasundaram R Washburn S Krzyspiak J Khodakhah K. Zona incerta modulation of the inferior olive and the pontine nuclei. Netw Neurosci. (2024) 8:260–74. doi: 10.1162/netn_a_00350 38562296 PMC10927296

[34] Masri R Quiton RL Lucas JM Murray PD Thompson SM Keller A. Zona incerta: a role in central pain. J Neurophysiol. (2009) 102:181–91. doi: 10.1152/jn.00152.2009 19403748 PMC2712264

[35] Holstege G. The periaqueductal gray controls brainstem emotional motor systems including respiration. Prog Brain Res. (2014) 209:379–405. doi: 10.1016/B978-0-444-63274-6.00020-5 24746059

[36] Siegel P Wepsic JG. Alteration of nociception by stimulation of cerebellar structures in the monkey. Physiol Behav. (1974) 13:189–94. doi: 10.1016/0031-9384(74)90033-X 4212925

[37] Saab CY Willis WD. Cerebellar stimulation modulates the intensity of a visceral nociceptive reflex in the rat. Exp Brain Res. (2002) 146:117–21. doi: 10.1007/s00221-002-1107-8 12192585

[38] Casey KL Minoshima S Berger KL Koeppe RA Morrow TJ Frey KA. Positron emission tomographic analysis of cerebral structures activated specifically by repetitive noxious heat stimuli. J Neurophysiol. (1994) 71:802–7. doi: 10.1152/jn.1994.71.2.802 8176441

[39] Helmchen C Mohr C Erdmann C Petersen D Nitschke MF. Differential cerebellar activation related to perceived pain intensity during noxious thermal stimulation in humans: a functional magnetic resonance imaging study. Neurosci Lett. (2003) 335:202–6. doi: 10.1016/S0304-3940(02)01164-3 12531467

[40] Helmchen C Mohr C Erdmann C Binkofski F. Cerebellar neural responses related to actively and passively applied noxious thermal stimulation in human subjects: a parametric fMRI study. Neurosci Lett. (2004) 361:237–40. doi: 10.1016/j.neulet.2003.12.017 15135937

[41] Basedau H May A Mehnert J. Cerebellar somatotopy of the trigemino-cervical complex during nociception. Eur J Pain. (2024) 28:719–28. doi: 10.1002/ejp.2212 38013614

[42] Schulte LH May A. The migraine generator revisited: continuous scanning of the migraine cycle over 30 days and three spontaneous attacks. Brain. (2016) 139:1987–93. doi: 10.1093/brain/aww097 27190019

[43] Stankewitz A Keidel L Rehm M Irving S Kaczmarz S Preibisch C . Migraine attacks as a result of hypothalamic loss of control. Neuroimage Clin. (2021) 32:102784. doi: 10.1016/j.nicl.2021.102784 34425551 PMC8379646

[44] Esteves I Fouto AR Ruiz-Tagle A Caetano G Nunes RG da Silva NA . Uncovering longitudinal changes in the brain functional connectome along the migraine cycle: a multilevel clinical connectome fingerprinting framework. J Headache Pain. (2025) 26:29. doi: 10.1186/s10194-025-01969-6 39930372 PMC11812190

[45] Afridi SK Matharu MS Lee L Kaube H Friston KJ Frackowiak RS . A PET study exploring the laterality of brainstem activation in migraine using glyceryl trinitrate. Brain. (2005) 128:932–9. doi: 10.1093/brain/awh416 15705611

[46] Schwedt TJ Chong CD Chiang CC Baxter L Schlaggar BL Dodick DW. Enhanced pain-induced activity of pain-processing regions in a case-control study of episodic migraine. Cephalalgia. (2014) 34:947–58. doi: 10.1177/0333102414526069 24627432 PMC4163130

[47] Chanraud S Di Scala G Dilharreguy B Schoenen J Allard M Radat F. Brain functional connectivity and morphology changes in medication-overuse headache: clue for dependence-related processes? Cephalalgia. (2014) 34:605–15. doi: 10.1177/0333102413519514 24449748

[48] Cao Z Yu W Zhang Z Xu M Lin J Zhang L . Decreased gray matter volume in the frontal cortex of migraine patients with associated functional connectivity alterations: a VBM and rs-FC study. Pain Res Manag. (2022) 2022:2115956. doi: 10.1155/2022/2115956 35126799 PMC8808241

[49] Gollion C Lerebours F Nemmi F Arribarat G Bonneville F Larrue V . Insular functional connectivity in migraine with aura. J Headache Pain. (2022) 23:106. doi: 10.1186/s10194-022-01473-1 35982396 PMC9389744

[50] Ke J Yu Y Zhang X Su Y Wang X Hu S . Functional alterations in the posterior insula and cerebellum in migraine without aura: a resting-state MRI study. Front Behav Neurosci. (2020) 14:567588. doi: 10.3389/fnbeh.2020.567588 33132860 PMC7573354

[51] Chen ZH Cui YL Sun JT Li YT Zhang C Zhang YM . The brain structure and function abnormalities of migraineurs: a systematic review and neuroimaging meta-analysis. Front Neurol. (2022) 13:1022793. doi: 10.3389/fneur.2022.1022793 36419535 PMC9676357

[52] Wang JJ Chen X Sah SK Zeng C Li YM Li N . Amplitude of low-frequency fluctuation (ALFF) and fractional ALFF in migraine patients: a resting-state functional MRI study. Clin Radiol. (2016) 71:558–64. doi: 10.1016/j.crad.2016.03.004 27055741

[53] Jia Z Yu S. Grey matter alterations in migraine: a systematic review and meta-analysis. Neuroimage Clin. (2017) 14:130–40. doi: 10.1016/j.nicl.2017.01.019 28180071 PMC5279908

[54] Masson R Demarquay G Meunier D Lévêque Y Hannoun S Bidet-Caulet A . Is migraine associated to brain anatomical alterations? New data and coordinate-based meta-analysis. Brain Topogr. (2021) 34:384–401. doi: 10.1007/s10548-021-00824-6 33606142

[55] Zhang X Zhou J Guo M Cheng S Chen Y Jiang N . A systematic review and meta-analysis of voxel-based morphometric studies of migraine. J Neurol. (2023) 270:152–70. doi: 10.1007/s00415-022-11363-w 36098838

[56] Al Qawasmeh M Ahmed YB Al-Bzour AN Al-Majali GN Alzghoul SM Al-Khalili AA . Meta-analytical evidence of functional and structural abnormalities associated with pain processing in migraine patients: an activation likelihood estimation. Medicine. (2022) 101:e31206. doi: 10.1097/MD.0000000000031206 36316871 PMC9622585

[57] Zhang J Wu YL Su J Yao Q Wang M Li GF . Assessment of gray and white matter structural alterations in migraineurs without aura. J Headache Pain. (2017) 18:74. doi: 10.1186/s10194-017-0783-5 28733941 PMC5520823

[58] Mehnert J May A. Functional and structural alterations in the migraine cerebellum. J Cereb Blood Flow Metab. (2019) 39:730–9. doi: 10.1177/0271678X17722109 28737061 PMC6446424

[59] Palm-Meinders IH Arkink EB Koppen H Amlal S Terwindt GM Launer LJ . Volumetric brain changes in migraineurs from the general population. Neurology. (2017) 89:2066–74. doi: 10.1212/WNL.0000000000004640 29021356 PMC5711510

[60] Aytaç A Aydin H Akay E. Multiplatform morphometric profiling of whole-brain, cerebellar subregional, and thalamic nuclei alterations in pediatric migraine without aura. Diagnostics. (2026) 16:2085. doi: 10.3390/diagnostics16132085 42449865 PMC13360080

[61] Messina R Rocca MA Colombo B Pagani E Falini A Goadsby PJ . Gray matter volume modifications in migraine: a cross-sectional and longitudinal study. Neurology. (2018) 91:e280–92. doi: 10.1212/WNL.0000000000005819 29925551

[62] Liu HY Lee PL Chou KH Lai KL Wang YF Chen SP . The cerebellum is associated with 2-year prognosis in patients with high-frequency migraine. J Headache Pain. (2020) 21:29. doi: 10.1186/s10194-020-01096-4 32188423 PMC7081533

[63] Rosell-Mirmi J Caronna E Gallardo VJ Pareto D Alpuente A Torres-Ferrús M . Brain structural MRI changes before and after 6 months of preventive treatment with erenumab and onabotulinumtoxinA in migraine. J Headache Pain. (2026) 27:175. doi: 10.1186/s10194-026-02393-0 42143217 PMC13348101

[64] Qin Z He XW Zhang J Xu S Li GF Su J . Structural changes of cerebellum and brainstem in migraine without aura. J Headache Pain. (2019) 20:93. doi: 10.1186/s10194-019-1045-5 31477012 PMC6734280

[65] Matoso A Fouto AR Esteves I Ruiz-Tagle A Caetano G da Silva NA . Involvement of the cerebellum in structural connectivity enhancement in episodic migraine. J Headache Pain. (2024) 25:154. doi: 10.1186/s10194-024-01854-8 39294590 PMC11409624

[66] Li Y Hu Y Dai C Fu F Cao Y Chen K . Abnormal structural connectivity-functional connectivity coupling beyond the cortex in patients with chronic migraine. J Headache Pain. (2026) 27:180. doi: 10.1186/s10194-026-02397-w 42157093 PMC13366957

[67] Kruit MC Launer LJ Ferrari MD van Buchem MA. Infarcts in the posterior circulation territory in migraine. The population-based MRI CAMERA study. Brain. (2005) 128:2068–77. doi: 10.1093/brain/awh542 16006538

[68] Kruit MC van Buchem MA Launer LJ Terwindt GM Ferrari MD. Migraine is associated with an increased risk of deep white matter lesions, subclinical posterior circulation infarcts and brain iron accumulation: the population-based MRI CAMERA study. Cephalalgia. (2010) 30:129–36. doi: 10.1111/j.1468-2982.2009.01904.x 19515125 PMC3241741

[69] Brighina F Palermo A Panetta ML Daniele O Aloisio A Cosentino G . Reduced cerebellar inhibition in migraine with aura: a TMS study. Cerebellum. (2009) 8:260–6. doi: 10.1007/s12311-008-0090-4 19156474

[70] Gerwig M Rauschen L Gaul C Katsarava Z Timmann D. Subclinical cerebellar dysfunction in patients with migraine: evidence from eyeblink conditioning. Cephalalgia. (2014) 34:904–13. doi: 10.1177/0333102414523844 24567118

[71] Koppen H Boele HJ Palm-Meinders IH Koutstaal BJ Horlings CG Koekkoek BKE . Cerebellar function and ischemic brain lesions in migraine patients from the general population. Cephalalgia. (2017) 37:177–90. doi: 10.1177/0333102416643527 27059879 PMC5852376

[72] May A. Experience-dependent structural plasticity in the adult human brain. Trends Cogn Sci. (2011) 15:475–82. doi: 10.1016/j.tics.2011.08.002 21906988

[73] Bilgiç B Kocaman G Arslan AB Noyan H Sherifov R Alkan A . Volumetric differences suggest involvement of cerebellum and brainstem in chronic migraine. Cephalalgia. (2016) 36:301–8. doi: 10.1177/0333102415588328 26002699

[74] Fumal A Laureys S Di Clemente L Boly M Bohotin V Vandenheede M . Orbitofrontal cortex involvement in chronic analgesic-overuse headache evolving from episodic migraine. Brain. (2006) 129:543–50. doi: 10.1093/brain/awh691 16330505

[75] Diener HC Dodick D Evers S Holle D Jensen RH Lipton RB . Pathophysiology, prevention, and treatment of medication overuse headache. Lancet Neurol. (2019) 18:891–902. doi: 10.1016/S1474-4422(19)30146-2 31174999

[76] Chen Z Chen X Chen Z Liu M He H Ma L . Alteration of gray matter texture features over the whole brain in medication-overuse headache using a 3-dimentional texture analysis. J Headache Pain. (2017) 18:112. doi: 10.1186/s10194-017-0820-4 29285575 PMC5745370

[77] Liu HY Yeh HH Lee PL Chen IJ Liao YH Chou KH . Distinct brain metabolic patterns reflect biological heterogeneity and relate to clinical outcome in chronic migraine with medication overuse. Cephalalgia. (2026) 46:3331024261476230. doi: 10.1177/03331024261476230 42578883

[78] Woolf CJ Salter MW. Neuronal plasticity: increasing the gain in pain. Science. (2000) 288:17651768. doi: 10.1126/science.288.5472.1765 10846153

[79] Edvinsson L Warfvinge K. Recognizing the role of CGRP and CGRP receptors in migraine and its treatment. Cephalalgia. (2019) 39:366–73. doi: 10.1177/0333102417736900 29020807

[80] Johnson KW Morin SM Wroblewski VJ Johnson MP. Peripheral and central nervous system distribution of the CGRP neutralizing antibody [^125^I] galcanezumab in male rats. Cephalalgia. (2019) 39:1241–8. doi: 10.1177/0333102419844711 31003588

[81] Pardridge WM. Drug transport across the blood-brain barrier. J Cereb Blood Flow Metab. (2012) 32:1959–72. doi: 10.1038/jcbfm.2012.126 22929442 PMC3494002

[82] Edvinsson L. CGRP receptor antagonists and antibodies against CGRP and its receptor in migraine treatment. Br J Clin Pharmacol. (2015) 80:193–9. doi: 10.1111/bcp.12618 25731075 PMC4541967

[83] Ziegeler C Mehnert J Asmussen K May A. Central effects of erenumab in migraine patients: An event-Related functional imaging study. Neurology. (2020) 95:e2794–802. doi: 10.1212/WNL.0000000000010740 32917805

[84] Filippi M Messina R Bartezaghi M Cetta I Colombo B Grazzi L . The effect of erenumab on brain network function in episodic migraine patients: a randomized, placebo-controlled clinical trial (RESET BRAIN). J Neurol. (2023) 270:5600–12. doi: 10.1007/s00415-023-11879-9 37550498 PMC10576673

[85] Basedau H Peng KP Schellong M May A. Double-blind, randomized, placebo-controlled study to evaluate erenumab-specific central effects: an fMRI study. J Headache Pain. (2024) 25:5. doi: 10.1186/s10194-023-01709-8 38195378 PMC10775481

[86] Ophoff RA Terwindt GM Vergouwe MN van Eijk R Oefner PJ Hoffman SM . Familial hemiplegic migraine and episodic ataxia type-2 are caused by mutations in the Ca2+ channel gene CACNL1A4. Cell. (1996) 87:543–52. doi: 10.1016/S0092-8674(00)81373-2 8898206

[87] Jen JC. Familial hemiplegic migraine. (2001) [Updated 2024 Jul 4]. In: GeneReviews^®^ [Internet]. Adam MP Bick S Mirzaa GM et al., editors. Seattle, WA: University of Washington. p. 1993–2026. Available online at: https://www.ncbi.nlm.nih.gov/books/NBK1388/ (Accessed August 25, 2026). 17395138

[88] Pietrobon D. CaV2. 1 channelopathies. Pflugers Arch. (2010) 460:375–93. doi: 10.1007/s00424-010-0802-8 20204399

[89] Lempert T Olesen J Furman J Waterston J Seemungal B Carey J . Vestibular migraine: diagnostic criteria. J Vestib Res. (2012) 22:167–72. doi: 10.3233/VES-2012-0453 23142830

[90] Shin JH Kim YK Kim HJ Kim JS. Altered brain metabolism in vestibular migraine: comparison of interictal and ictal findings. Cephalalgia. (2014) 34:58–67. doi: 10.1177/0333102413498940 23918837

[91] Xiong X Dai L Chen W Lu J Hu C Zhao H . Dynamics and concordance alterations of regional brain function indices in vestibular migraine: a resting-state fMRI study. J Headache Pain. (2024) 25:1. doi: 10.1186/s10194-023-01705-y 38178029 PMC10768112

[92] Chen W Zhao H Feng Q Xiong X Ke J Dai L . Disrupted gray matter connectome in vestibular migraine: a combined machine learning and individual-level morphological brain network analysis. J Headache Pain. (2024) 25:177. doi: 10.1186/s10194-024-01861-9 39390381 PMC11468853

[93] Zhe X Zhang X Chen L Zhang L Tang M Zhang D . Altered gray matter volume and functional connectivity in patients with vestibular migraine. Front Neurosci. (2021) 15:683802. doi: 10.3389/fnins.2021.683802 34305518 PMC8297163

[94] Puledda F Bruchhage M O'Daly O Ffytche D Williams SCR Goadsby PJ. Occipital cortex and cerebellum gray matter changes in visual snow syndrome. Neurology. (2020) 95:e1792–9. doi: 10.1212/WNL.0000000000010530 32759201 PMC7682819

[95] Puledda F O'Daly O Schankin C Ffytche D Williams SC Goadsby PJ. Disrupted connectivity within visual, attentional and salience networks in the visual snow syndrome. Hum Brain Mapp. (2021) 42:2032–44. doi: 10.1002/hbm.25343 33448525 PMC8046036

[96] Silva EM Puledda F. Visual snow syndrome and migraine: a review. Eye. (2023) 37:23742378. doi: 10.1038/s41433-023-02435-w 36788360 PMC10397188

[97] Koch G Mori F Marconi B Codecà C Pecchioli C Salerno S . Changes in intracortical circuits of the human motor cortex following theta burst stimulation of the lateral cerebellum. Clin Neurophysiol. (2008) 119:2559–69. doi: 10.1016/j.clinph.2008.08.008 18824403

[98] Nitsche MA Paulus W. Excitability changes induced in the human motor cortex by weak transcranial direct current stimulation. J Physiol. (2000) 527:633–9. doi: 10.1111/j.1469-7793.2000.t01-1-00633.x 10990547 PMC2270099

[99] Betancur DFA da Graça Lopes Tarragó M França BR Bueno GB Gomes J Coelho ML . Cortical excitability and analgesic effects of multisite transcranial direct current stimulation targeting the motor cortex and cerebellum in fibromyalgia. Sci Rep. (2025) 15:37067. doi: 10.1038/s41598-025-20769-1 41130992 PMC12549915

[100] Schading S Pohl H Gantenbein A Luechinger R Sandor P Riederer F . Tracking tDCS induced grey matter changes in episodic migraine: a randomized controlled trial. J Headache Pain. (2021) 22:139. doi: 10.1186/s10194-021-01347-y 34800989 PMC8605508

